# 4-(1,3-Diphenyl-4,5-dihydro-1*H*-pyrazol-5-yl)-1,3-diphenyl-1*H*-pyrazole

**DOI:** 10.1107/S1600536811039869

**Published:** 2011-10-05

**Authors:** Hoong-Kun Fun, Tze Shyang Chia, Shridhar Malladi, Arun M. Isloor, Kammasandra N. Shivananda

**Affiliations:** a X-ray Crystallography Unit, School of Physics, Universiti Sains Malaysia, 11800 USM, Penang, Malaysia; b Medicinal Chemistry Section, Department of Chemistry, National Institute of Technology–Karnataka, Surathkal, Mangalore 575 025, India; c Schulich Faculty of Chemistry, Technion Israel Institute of Technology, Haifa 32000, Israel

## Abstract

The title compound, C_30_H_24_N_4_, contains two pyrazole rings and four phenyl rings. The pyrazole rings are essentially planar, with maximum deviations of 0.003 (1) and 0.066 (1) Å and make a dihedral angle of 73.43 (6)°.  The two pyrazole rings make dihedral angles of 40.08 (6), 9.28 (6), 15.78 (8) and 17.25 (7)° with their attached phenyl rings. In the crystal, there are no significant inter­molecular hydrogen-bonding inter­actions. The crystal structure is stabilized by C—H⋯π inter­actions.

## Related literature

For the pharmacological activity of substituted 2-pyrazolines, see: Sahu *et al.* (2008 ▶); Farghaly *et al.* (1990 ▶); Adnan *et al.* (2005 ▶); Budakoti *et al.* (2008 ▶); Yar *et al.* (2007 ▶); Palaska *et al.* (1996 ▶); Jia *et al.* (2004 ▶). For the experimental preparation, see: Bratenko *et al.* (2001 ▶). For related structures, see: Fun *et al.* (2010 ▶, 2011 ▶). For reference bond lengths, see: Allen *et al.* (1987 ▶).
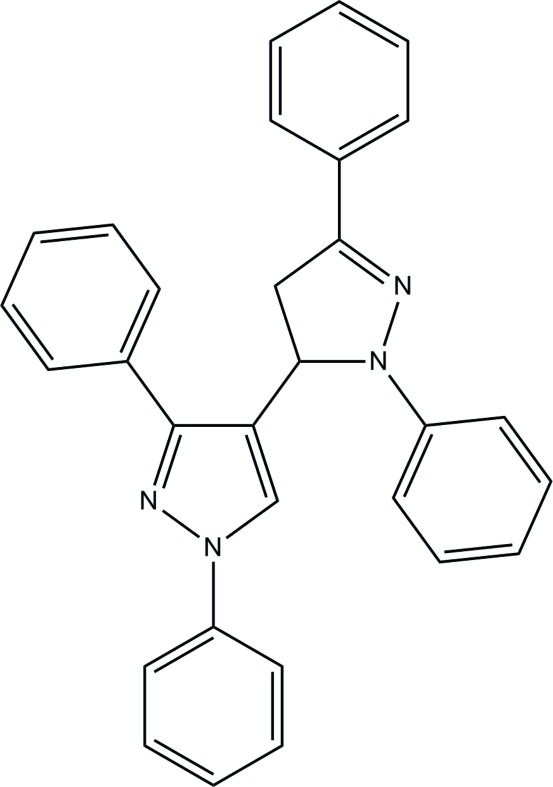


## Experimental

### 

#### Crystal data

C_30_H_24_N_4_
*M* *_r_* = 440.53
Monoclinic, 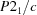

*a* = 10.7841 (5) Å
*b* = 11.0582 (6) Å
*c* = 21.4820 (9) Å
β = 113.359 (2)°
*V* = 2351.82 (19) Å^3^
*Z* = 4
Mo *K*α radiation
μ = 0.08 mm^−1^
*T* = 296 K
0.56 × 0.54 × 0.36 mm

#### Data collection

Bruker APEX DUO CCD area-detector diffractometer
Absorption correction: multi-scan (*SADABS*; Bruker, 2009 ▶) *T*_min_ = 0.960, *T*_max_ = 0.974
22465 measured reflections
7042 independent reflections
5057 reflections with *I* > 2σ(*I*)
*R*_int_ = 0.020

#### Refinement

*R*[*F* ^2^ > 2σ(*F* ^2^)] = 0.043
*wR*(*F* ^2^) = 0.120
*S* = 1.01
7042 reflections
307 parameters
H-atom parameters constrained
Δρ_max_ = 0.19 e Å^−3^
Δρ_min_ = −0.17 e Å^−3^

### 

Data collection: *APEX2* (Bruker, 2009 ▶); cell refinement: *SAINT* (Bruker, 2009 ▶); data reduction: *SAINT*; program(s) used to solve structure: *SHELXTL* (Sheldrick, 2008 ▶); program(s) used to refine structure: *SHELXTL*; molecular graphics: *SHELXTL*; software used to prepare material for publication: *SHELXTL* and *PLATON* (Spek, 2009 ▶).

## Supplementary Material

Crystal structure: contains datablock(s) global, I. DOI: 10.1107/S1600536811039869/kj2190sup1.cif

Structure factors: contains datablock(s) I. DOI: 10.1107/S1600536811039869/kj2190Isup2.hkl

Supplementary material file. DOI: 10.1107/S1600536811039869/kj2190Isup3.cml

Additional supplementary materials:  crystallographic information; 3D view; checkCIF report

## Figures and Tables

**Table 1:** Hydrogen-bond geometry (Å, °) *Cg*1 is the centroid of the C1–C6 ring.

*D*—H⋯*A*	*D*—H	H⋯*A*	*D*⋯*A*	*D*—H⋯*A*
C8—H8*A*⋯*Cg*1^i^	0.97	2.95	3.6999 (15)	135

Symmetry code: (i) 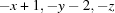.

**Fig. 1.**
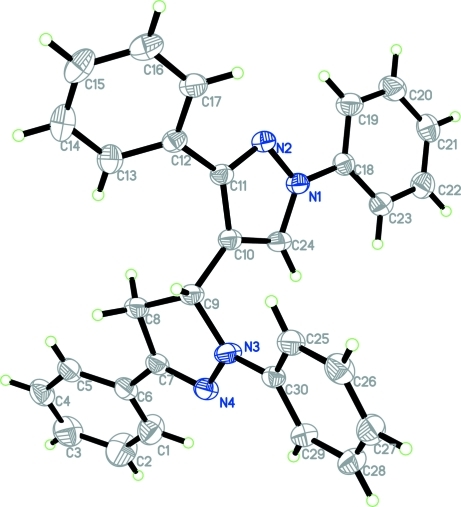
The molecular structure of the title compound with atom labels with 30% probability displacement ellipsoids.

## supplementary crystallographic information

### Comment

Pyrazolines are nitrogen-containing five-membered heterocyclic compounds and have received considerable attention in recent years due to their varied biological and pharmacological activities. Various substituted 2-pyrazolines have been associated with diverse pharmacological activities such as analgesic (Sahu *et al.*, 2008), anti-inflammatory (Farghaly *et al.*, 1990), anti-microbial (Adnan *et al.*, 2005), anti-amoebic (Budakoti *et al.*, 2008), anti-tubercular (Yar *et al.*, 2007), anti-depressant (Palaska *et al.*, 1996) and anti-coagulant (Jia *et al.*, 2004) properties. Based on the above biological activities exhibited by the pyrazolines, we have synthesized the title compound to study its crystal structure.

The molecular structure of the title compound, shown in Fig. 1, contains two pyrazole (N1,N2/C10,C11,C24) and (N3,N4/C7–C9) rings and four phenyl (C1–C6), (C12–C17), (C18–C23) and (C25–C30) rings. The pyrazole rings are essentially planar with maximum deviation of 0.003 (1) Å for atom C10 and 0.066 (1) Å for atom C9. The two pyrazole (N1,N2/C10,C11,C24:N3,N4/C7–C9) rings make dihedral angles of 40.08 (6), 9.28 (6), 15.78 (8) and 17.25 (7)° with their attached phenyl (C12–C17/C18–C23):(C1–C6/C25–C30) rings respectively. The dihedral angle between the two pyrazole, (N1,N2/C10,C11,C24: N3,N4/C7–C9), rings is 73.43 (6)°. Bond lengths (Allen *et al.*, 1987) and angles are within normal ranges and are comparable to related structures (Fun *et al.*, 2010; Fun *et al.*, 2011).

There are no significant intermolecular hydrogen bond interactions in the crystal structure. The structure is stabilized by C8—H8A···*Cg*1 (Table 1) interactions where *Cg*1 is the centroid of the C1–C6 ring.

### Experimental

A mixture of (2*E*)-3-(1,3-diphenyl-1*H*-pyrazol-4-yl)-1-phenylprop- 2-en-1-one (0.35 g, 1.0 mmol) and phenylhydrazine (0.162 g, 1.5 mmol) was refluxed in glacial acetic acid for 4 h. The mixture was then cooled to room temperature and the resulting solid was filtered and dried to get title compound. Yield: 0.22 g, 50%. M. p. 467–469 K (Bratenko *et al.*, 2001).

### Refinement

All H atoms were positioned geometrically [C—H = 0.93–0.98 Å] and refined using a riding model with *U*_iso_(H) = 1.2 *U*_eq_(C).

### Crystal data

**Table:**

C_30_H_24_N_4_	*F*(000) = 928
*M**_r_* = 440.53	*D*_x_ = 1.244 Mg m^−^^3^
Monoclinic, *P*2_1_/*c*	Mo *K*α radiation, λ = 0.71073 Å
Hall symbol: -P 2ybc	Cell parameters from 6778 reflections
*a* = 10.7841 (5) Å	θ = 2.9–30.3°
*b* = 11.0582 (6) Å	µ = 0.08 mm^−^^1^
*c* = 21.4820 (9) Å	*T* = 296 K
β = 113.359 (2)°	Block, colourless
*V* = 2351.82 (19) Å^3^	0.56 × 0.54 × 0.36 mm
*Z* = 4	

### Data collection

**Table:**

Bruker APEX DUO CCD area-detector diffractometer	7042 independent reflections
Radiation source: fine-focus sealed tube	5057 reflections with *I* > 2σ(*I*)
graphite	*R*_int_ = 0.020
φ and ω scans	θ_max_ = 30.4°, θ_min_ = 2.1°
Absorption correction: multi-scan (*SADABS*; Bruker, 2009)	*h* = −14→15
*T*_min_ = 0.960, *T*_max_ = 0.974	*k* = −15→15
22465 measured reflections	*l* = −30→28

### Refinement

**Table:**

Refinement on *F*^2^	Primary atom site location: structure-invariant direct methods
Least-squares matrix: full	Secondary atom site location: difference Fourier map
*R*[*F*^2^ > 2σ(*F*^2^)] = 0.043	Hydrogen site location: inferred from neighbouring sites
*wR*(*F*^2^) = 0.120	H-atom parameters constrained
*S* = 1.01	*w* = 1/[σ^2^(*F*_o_^2^) + (0.0521*P*)^2^ + 0.329*P*] where *P* = (*F*_o_^2^ + 2*F*_c_^2^)/3
7042 reflections	(Δ/σ)_max_ = 0.001
307 parameters	Δρ_max_ = 0.19 e Å^−^^3^
0 restraints	Δρ_min_ = −0.17 e Å^−^^3^

### Special details

**Table:**

Geometry. All e.s.d.'s (except the e.s.d. in the dihedral angle between two l.s. planes) are estimated using the full covariance matrix. The cell e.s.d.'s are taken into account individually in the estimation of e.s.d.'s in distances, angles and torsion angles; correlations between e.s.d.'s in cell parameters are only used when they are defined by crystal symmetry. An approximate (isotropic) treatment of cell e.s.d.'s is used for estimating e.s.d.'s involving l.s. planes.
Refinement. Refinement of *F*^2^ against ALL reflections. The weighted *R*-factor *wR* and goodness of fit *S* are based on *F*^2^, conventional *R*-factors *R* are based on *F*, with *F* set to zero for negative *F*^2^. The threshold expression of *F*^2^ > σ(*F*^2^) is used only for calculating *R*-factors(gt) *etc*. and is not relevant to the choice of reflections for refinement. *R*-factors based on *F*^2^ are statistically about twice as large as those based on *F*, and *R*- factors based on ALL data will be even larger.

### Fractional atomic coordinates and isotropic or equivalent isotropic displacement parameters (Å^2^)

**Table:**

	*x*	*y*	*z*	*U*_iso_*/*U*_eq_	
N1	0.94191 (8)	0.14967 (8)	0.85152 (4)	0.0419 (2)	
N2	1.01177 (8)	0.04544 (8)	0.87428 (4)	0.0420 (2)	
N3	0.56225 (8)	0.05489 (10)	0.84967 (4)	0.0491 (2)	
N4	0.51695 (9)	0.12835 (9)	0.88820 (5)	0.0455 (2)	
C1	0.49306 (18)	0.27780 (16)	0.99429 (8)	0.0766 (4)	
H1A	0.4525	0.3083	0.9505	0.092*	
C2	0.4751 (2)	0.3365 (2)	1.04702 (9)	0.0983 (6)	
H2A	0.4230	0.4063	1.0385	0.118*	
C3	0.53416 (19)	0.29191 (19)	1.11214 (8)	0.0857 (5)	
H3A	0.5219	0.3315	1.1475	0.103*	
C4	0.61059 (15)	0.18962 (16)	1.12451 (7)	0.0681 (4)	
H4A	0.6501	0.1592	1.1684	0.082*	
C5	0.62987 (12)	0.13063 (13)	1.07223 (6)	0.0548 (3)	
H5A	0.6828	0.0612	1.0814	0.066*	
C6	0.57077 (11)	0.17416 (12)	1.00623 (6)	0.0493 (3)	
C7	0.59097 (10)	0.10939 (11)	0.95147 (5)	0.0442 (2)	
C8	0.69495 (11)	0.01206 (12)	0.96309 (6)	0.0495 (3)	
H8A	0.6731	−0.0596	0.9828	0.059*	
H8B	0.7843	0.0401	0.9924	0.059*	
C9	0.68557 (10)	−0.01291 (11)	0.89089 (5)	0.0439 (2)	
H9A	0.6726	−0.0995	0.8807	0.053*	
C10	0.80458 (10)	0.03321 (10)	0.87806 (5)	0.0410 (2)	
C11	0.92896 (10)	−0.02591 (10)	0.89020 (5)	0.0396 (2)	
C12	0.97411 (10)	−0.14903 (10)	0.91534 (5)	0.0419 (2)	
C13	0.94926 (13)	−0.19930 (12)	0.96866 (6)	0.0544 (3)	
H13A	0.8983	−0.1568	0.9876	0.065*	
C14	1.00005 (17)	−0.31211 (13)	0.99366 (7)	0.0673 (4)	
H14A	0.9834	−0.3449	1.0295	0.081*	
C15	1.07492 (15)	−0.37602 (13)	0.96598 (7)	0.0671 (4)	
H15A	1.1089	−0.4518	0.9831	0.080*	
C16	1.09970 (13)	−0.32790 (12)	0.91289 (7)	0.0615 (3)	
H16A	1.1506	−0.3711	0.8942	0.074*	
C17	1.04886 (11)	−0.21513 (11)	0.88733 (6)	0.0504 (3)	
H17A	1.0649	−0.1834	0.8511	0.061*	
C18	1.00229 (11)	0.24748 (10)	0.83059 (5)	0.0429 (2)	
C19	1.13800 (13)	0.24282 (13)	0.84213 (7)	0.0610 (3)	
H19A	1.1901	0.1762	0.8636	0.073*	
C20	1.19517 (14)	0.33842 (14)	0.82140 (8)	0.0667 (4)	
H20A	1.2862	0.3354	0.8290	0.080*	
C21	1.12019 (15)	0.43753 (13)	0.78986 (7)	0.0619 (3)	
H21A	1.1598	0.5011	0.7761	0.074*	
C22	0.98606 (16)	0.44169 (13)	0.77892 (7)	0.0657 (4)	
H22A	0.9346	0.5088	0.7578	0.079*	
C23	0.92646 (13)	0.34718 (12)	0.79900 (6)	0.0561 (3)	
H23A	0.8353	0.3508	0.7912	0.067*	
C24	0.81716 (10)	0.14420 (11)	0.85323 (5)	0.0447 (2)	
H24A	0.7523	0.2050	0.8399	0.054*	
C25	0.51983 (12)	−0.05529 (12)	0.74638 (6)	0.0519 (3)	
H25A	0.5994	−0.0987	0.7678	0.062*	
C26	0.43826 (13)	−0.07908 (14)	0.67931 (6)	0.0601 (3)	
H26A	0.4622	−0.1400	0.6564	0.072*	
C27	0.32226 (13)	−0.01377 (15)	0.64623 (6)	0.0661 (4)	
H27A	0.2683	−0.0298	0.6010	0.079*	
C28	0.28672 (12)	0.07572 (15)	0.68070 (6)	0.0634 (4)	
H28A	0.2087	0.1206	0.6583	0.076*	
C29	0.36529 (11)	0.09971 (12)	0.74803 (6)	0.0511 (3)	
H29A	0.3396	0.1599	0.7708	0.061*	
C30	0.48330 (10)	0.03349 (10)	0.78193 (5)	0.0417 (2)	

### Atomic displacement parameters (Å^2^)

**Table:**

	*U*^11^	*U*^22^	*U*^33^	*U*^12^	*U*^13^	*U*^23^
N1	0.0377 (4)	0.0422 (5)	0.0463 (4)	0.0033 (4)	0.0172 (3)	0.0007 (4)
N2	0.0369 (4)	0.0416 (5)	0.0467 (4)	0.0037 (4)	0.0158 (3)	0.0003 (4)
N3	0.0339 (4)	0.0674 (7)	0.0420 (4)	0.0082 (4)	0.0110 (3)	−0.0068 (4)
N4	0.0390 (4)	0.0527 (6)	0.0462 (5)	−0.0005 (4)	0.0186 (4)	−0.0038 (4)
C1	0.0935 (11)	0.0828 (11)	0.0567 (7)	0.0240 (9)	0.0331 (7)	0.0010 (7)
C2	0.1249 (16)	0.1000 (14)	0.0798 (11)	0.0404 (12)	0.0507 (11)	−0.0055 (10)
C3	0.0965 (12)	0.1080 (14)	0.0648 (9)	0.0062 (11)	0.0450 (9)	−0.0180 (9)
C4	0.0662 (8)	0.0947 (11)	0.0492 (7)	−0.0118 (8)	0.0289 (6)	−0.0060 (7)
C5	0.0488 (6)	0.0685 (8)	0.0501 (6)	−0.0091 (6)	0.0230 (5)	−0.0019 (6)
C6	0.0452 (6)	0.0596 (7)	0.0473 (6)	−0.0063 (5)	0.0230 (5)	−0.0048 (5)
C7	0.0372 (5)	0.0518 (6)	0.0456 (5)	−0.0070 (4)	0.0186 (4)	−0.0025 (5)
C8	0.0356 (5)	0.0677 (8)	0.0440 (5)	0.0006 (5)	0.0146 (4)	0.0007 (5)
C9	0.0318 (4)	0.0536 (6)	0.0442 (5)	0.0019 (4)	0.0129 (4)	−0.0013 (5)
C10	0.0337 (4)	0.0474 (6)	0.0404 (5)	0.0011 (4)	0.0132 (4)	−0.0033 (4)
C11	0.0338 (4)	0.0442 (6)	0.0388 (5)	0.0004 (4)	0.0121 (4)	−0.0027 (4)
C12	0.0336 (4)	0.0435 (6)	0.0426 (5)	−0.0026 (4)	0.0086 (4)	−0.0017 (4)
C13	0.0614 (7)	0.0534 (7)	0.0468 (6)	−0.0032 (6)	0.0198 (5)	−0.0020 (5)
C14	0.0897 (10)	0.0547 (8)	0.0492 (6)	−0.0078 (7)	0.0187 (6)	0.0067 (6)
C15	0.0713 (8)	0.0439 (7)	0.0641 (8)	0.0017 (6)	0.0036 (6)	0.0054 (6)
C16	0.0517 (7)	0.0503 (7)	0.0751 (8)	0.0077 (6)	0.0171 (6)	−0.0033 (6)
C17	0.0425 (5)	0.0485 (6)	0.0598 (7)	0.0032 (5)	0.0197 (5)	0.0025 (5)
C18	0.0463 (5)	0.0430 (6)	0.0411 (5)	−0.0002 (5)	0.0191 (4)	−0.0030 (4)
C19	0.0487 (6)	0.0565 (8)	0.0801 (9)	0.0042 (6)	0.0281 (6)	0.0130 (7)
C20	0.0558 (7)	0.0690 (9)	0.0812 (9)	−0.0076 (7)	0.0334 (7)	0.0055 (7)
C21	0.0755 (9)	0.0559 (8)	0.0617 (7)	−0.0086 (7)	0.0350 (7)	0.0027 (6)
C22	0.0761 (9)	0.0554 (8)	0.0710 (8)	0.0095 (7)	0.0349 (7)	0.0169 (7)
C23	0.0534 (6)	0.0566 (7)	0.0602 (7)	0.0079 (6)	0.0247 (5)	0.0097 (6)
C24	0.0367 (5)	0.0479 (6)	0.0493 (5)	0.0064 (4)	0.0168 (4)	−0.0003 (5)
C25	0.0448 (6)	0.0620 (8)	0.0486 (6)	0.0007 (5)	0.0182 (5)	−0.0040 (5)
C26	0.0602 (7)	0.0720 (9)	0.0511 (6)	−0.0144 (6)	0.0255 (6)	−0.0142 (6)
C27	0.0547 (7)	0.0906 (11)	0.0434 (6)	−0.0177 (7)	0.0093 (5)	−0.0051 (6)
C28	0.0436 (6)	0.0807 (10)	0.0535 (7)	−0.0010 (6)	0.0062 (5)	0.0092 (7)
C29	0.0395 (5)	0.0578 (7)	0.0522 (6)	0.0019 (5)	0.0141 (5)	0.0022 (5)
C30	0.0326 (4)	0.0514 (6)	0.0407 (5)	−0.0046 (4)	0.0141 (4)	−0.0004 (4)

### Geometric parameters (Å, °)

**Table:**

N1—N2	1.3576 (12)	C13—H13A	0.9300
N1—C24	1.3617 (14)	C14—C15	1.373 (2)
N1—C18	1.4241 (14)	C14—H14A	0.9300
N2—C11	1.3339 (14)	C15—C16	1.377 (2)
N3—N4	1.3798 (13)	C15—H15A	0.9300
N3—C30	1.3843 (13)	C16—C17	1.3851 (18)
N3—C9	1.4768 (13)	C16—H16A	0.9300
N4—C7	1.2917 (14)	C17—H17A	0.9300
C1—C6	1.382 (2)	C18—C23	1.3801 (16)
C1—C2	1.384 (2)	C18—C19	1.3850 (16)
C1—H1A	0.9300	C19—C20	1.3834 (19)
C2—C3	1.378 (3)	C19—H19A	0.9300
C2—H2A	0.9300	C20—C21	1.371 (2)
C3—C4	1.362 (3)	C20—H20A	0.9300
C3—H3A	0.9300	C21—C22	1.371 (2)
C4—C5	1.3839 (19)	C21—H21A	0.9300
C4—H4A	0.9300	C22—C23	1.3826 (19)
C5—C6	1.3899 (17)	C22—H22A	0.9300
C5—H5A	0.9300	C23—H23A	0.9300
C6—C7	1.4653 (16)	C24—H24A	0.9300
C7—C8	1.5021 (17)	C25—C26	1.3835 (17)
C8—C9	1.5388 (15)	C25—C30	1.3931 (17)
C8—H8A	0.9700	C25—H25A	0.9300
C8—H8B	0.9700	C26—C27	1.373 (2)
C9—C10	1.5045 (15)	C26—H26A	0.9300
C9—H9A	0.9800	C27—C28	1.378 (2)
C10—C24	1.3663 (16)	C27—H27A	0.9300
C10—C11	1.4202 (14)	C28—C29	1.3814 (17)
C11—C12	1.4743 (15)	C28—H28A	0.9300
C12—C17	1.3895 (16)	C29—C30	1.3968 (15)
C12—C13	1.3914 (16)	C29—H29A	0.9300
C13—C14	1.382 (2)		
			
N2—N1—C24	111.45 (9)	C12—C13—H13A	119.9
N2—N1—C18	120.04 (8)	C15—C14—C13	120.44 (13)
C24—N1—C18	128.50 (9)	C15—C14—H14A	119.8
C11—N2—N1	105.20 (8)	C13—C14—H14A	119.8
N4—N3—C30	120.98 (8)	C14—C15—C16	120.01 (13)
N4—N3—C9	112.84 (8)	C14—C15—H15A	120.0
C30—N3—C9	125.15 (9)	C16—C15—H15A	120.0
C7—N4—N3	108.52 (9)	C15—C16—C17	119.98 (13)
C6—C1—C2	120.52 (15)	C15—C16—H16A	120.0
C6—C1—H1A	119.7	C17—C16—H16A	120.0
C2—C1—H1A	119.7	C16—C17—C12	120.56 (12)
C3—C2—C1	120.35 (17)	C16—C17—H17A	119.7
C3—C2—H2A	119.8	C12—C17—H17A	119.7
C1—C2—H2A	119.8	C23—C18—C19	119.72 (11)
C4—C3—C2	119.68 (15)	C23—C18—N1	120.40 (10)
C4—C3—H3A	120.2	C19—C18—N1	119.88 (10)
C2—C3—H3A	120.2	C20—C19—C18	119.22 (12)
C3—C4—C5	120.46 (14)	C20—C19—H19A	120.4
C3—C4—H4A	119.8	C18—C19—H19A	120.4
C5—C4—H4A	119.8	C21—C20—C19	121.28 (13)
C4—C5—C6	120.60 (14)	C21—C20—H20A	119.4
C4—C5—H5A	119.7	C19—C20—H20A	119.4
C6—C5—H5A	119.7	C20—C21—C22	119.14 (13)
C1—C6—C5	118.38 (12)	C20—C21—H21A	120.4
C1—C6—C7	121.90 (11)	C22—C21—H21A	120.4
C5—C6—C7	119.71 (12)	C21—C22—C23	120.70 (13)
N4—C7—C6	122.61 (11)	C21—C22—H22A	119.7
N4—C7—C8	113.59 (10)	C23—C22—H22A	119.7
C6—C7—C8	123.76 (10)	C18—C23—C22	119.95 (12)
C7—C8—C9	102.40 (9)	C18—C23—H23A	120.0
C7—C8—H8A	111.3	C22—C23—H23A	120.0
C9—C8—H8A	111.3	N1—C24—C10	107.60 (9)
C7—C8—H8B	111.3	N1—C24—H24A	126.2
C9—C8—H8B	111.3	C10—C24—H24A	126.2
H8A—C8—H8B	109.2	C26—C25—C30	120.24 (12)
N3—C9—C10	110.42 (9)	C26—C25—H25A	119.9
N3—C9—C8	101.35 (8)	C30—C25—H25A	119.9
C10—C9—C8	113.82 (9)	C27—C26—C25	120.76 (13)
N3—C9—H9A	110.3	C27—C26—H26A	119.6
C10—C9—H9A	110.3	C25—C26—H26A	119.6
C8—C9—H9A	110.3	C26—C27—C28	119.33 (11)
C24—C10—C11	104.63 (9)	C26—C27—H27A	120.3
C24—C10—C9	126.87 (10)	C28—C27—H27A	120.3
C11—C10—C9	128.49 (10)	C27—C28—C29	120.97 (12)
N2—C11—C10	111.11 (10)	C27—C28—H28A	119.5
N2—C11—C12	118.86 (9)	C29—C28—H28A	119.5
C10—C11—C12	130.02 (10)	C28—C29—C30	119.94 (12)
C17—C12—C13	118.71 (11)	C28—C29—H29A	120.0
C17—C12—C11	119.42 (10)	C30—C29—H29A	120.0
C13—C12—C11	121.80 (10)	N3—C30—C25	120.47 (10)
C14—C13—C12	120.29 (13)	N3—C30—C29	120.79 (10)
C14—C13—H13A	119.9	C25—C30—C29	118.74 (10)
			
C24—N1—N2—C11	0.15 (11)	N2—C11—C12—C13	138.80 (11)
C18—N1—N2—C11	179.35 (9)	C10—C11—C12—C13	−42.50 (16)
C30—N3—N4—C7	163.87 (10)	C17—C12—C13—C14	0.96 (17)
C9—N3—N4—C7	−5.10 (13)	C11—C12—C13—C14	−176.08 (11)
C6—C1—C2—C3	−0.3 (3)	C12—C13—C14—C15	−0.3 (2)
C1—C2—C3—C4	0.1 (3)	C13—C14—C15—C16	−0.1 (2)
C2—C3—C4—C5	0.3 (3)	C14—C15—C16—C17	−0.1 (2)
C3—C4—C5—C6	−0.5 (2)	C15—C16—C17—C12	0.78 (19)
C2—C1—C6—C5	0.1 (2)	C13—C12—C17—C16	−1.18 (17)
C2—C1—C6—C7	179.55 (16)	C11—C12—C17—C16	175.93 (10)
C4—C5—C6—C1	0.28 (19)	N2—N1—C18—C23	171.28 (10)
C4—C5—C6—C7	−179.17 (11)	C24—N1—C18—C23	−9.67 (17)
N3—N4—C7—C6	179.29 (10)	N2—N1—C18—C19	−8.90 (15)
N3—N4—C7—C8	−2.93 (13)	C24—N1—C18—C19	170.15 (12)
C1—C6—C7—N4	−15.00 (19)	C23—C18—C19—C20	−0.3 (2)
C5—C6—C7—N4	164.43 (11)	N1—C18—C19—C20	179.88 (12)
C1—C6—C7—C8	167.43 (13)	C18—C19—C20—C21	0.2 (2)
C5—C6—C7—C8	−13.13 (17)	C19—C20—C21—C22	0.2 (2)
N4—C7—C8—C9	9.08 (13)	C20—C21—C22—C23	−0.3 (2)
C6—C7—C8—C9	−173.16 (10)	C19—C18—C23—C22	0.14 (19)
N4—N3—C9—C10	−110.71 (10)	N1—C18—C23—C22	179.96 (12)
C30—N3—C9—C10	80.87 (13)	C21—C22—C23—C18	0.2 (2)
N4—N3—C9—C8	10.24 (12)	N2—N1—C24—C10	0.20 (12)
C30—N3—C9—C8	−158.19 (11)	C18—N1—C24—C10	−178.92 (10)
C7—C8—C9—N3	−10.62 (11)	C11—C10—C24—N1	−0.44 (11)
C7—C8—C9—C10	107.91 (10)	C9—C10—C24—N1	178.21 (10)
N3—C9—C10—C24	22.18 (15)	C30—C25—C26—C27	1.7 (2)
C8—C9—C10—C24	−91.02 (13)	C25—C26—C27—C28	−0.5 (2)
N3—C9—C10—C11	−159.50 (10)	C26—C27—C28—C29	−0.6 (2)
C8—C9—C10—C11	87.30 (14)	C27—C28—C29—C30	0.6 (2)
N1—N2—C11—C10	−0.43 (11)	N4—N3—C30—C25	−169.65 (11)
N1—N2—C11—C12	178.50 (8)	C9—N3—C30—C25	−2.11 (17)
C24—C10—C11—N2	0.55 (12)	N4—N3—C30—C29	9.94 (17)
C9—C10—C11—N2	−178.06 (10)	C9—N3—C30—C29	177.48 (11)
C24—C10—C11—C12	−178.22 (10)	C26—C25—C30—N3	177.86 (11)
C9—C10—C11—C12	3.16 (18)	C26—C25—C30—C29	−1.74 (18)
N2—C11—C12—C17	−38.22 (14)	C28—C29—C30—N3	−178.99 (12)
C10—C11—C12—C17	140.48 (11)	C28—C29—C30—C25	0.61 (18)

### Hydrogen-bond geometry (Å, °)

**Table:**

*Cg*1 is the centroid of the C1–C6 ring.

**Table:**

*D*—H···*A*	*D*—H	H···*A*	*D*···*A*	*D*—H···*A*
C8—H8A···Cg1^i^	0.97	2.95	3.6999 (15)	135

Symmetry codes: (i) −*x*+1, −*y*−2, −*z*.
